# Research hotspots and trends for axon regeneration (2000–2021): a bibliometric study and systematic review

**DOI:** 10.1186/s41232-022-00244-4

**Published:** 2022-12-07

**Authors:** Yuyu Chou, Homaira Nawabi, Jingze Li

**Affiliations:** 1 Department of Ophthalmology, Peking Union Medical College Hospital, Chinese Academy of Medical Sciences & Peking Union Medical College, Beijing, 100730 China; 2 Grenoble Institut Neurosciences, Inserm, U1216, Grenoble Alpes University, Grenoble, France; 3 Key Laboratory of Metallogenic Prediction of Nonferrous Metals and Geological Environment Monitoring, Ministry of Education, School of Geosciences and Info-Physics, Central South University, Changsha, 410083 People’s Republic of China; 4 Laboratory 3SR, Grenoble Alpes University, CNRS UMR 5521, 38400 Grenoble, France

**Keywords:** Axon regeneration, Bibliometric analysis, Latent Dirichlet Allocation analysis, Neuronal intrinsic pathways, Extrinsic environment, Multifactor regulations

## Abstract

**Background:**

Axons play an essential role in the connection of the nervous system with the rest of the body. Axon lesions often lead to permanent impairment of motor and cognitive functions and the interaction with the outside world. Studies focusing on axon regeneration have become a research field with considerable interest. The purpose of this study is to obtain an overall perspective of the research field of axonal regeneration and to assist the researchers and the funding agencies to better know the areas of greatest research opportunities.

**Methods:**

We conducted a bibliometric analysis and Latent Dirichlet Allocation (LDA) analysis of the global literature on axon regeneration based on the Web of Science (WoS) over the recent 22 years, to address the research hotspots, publication trends, and understudied areas.

**Results:**

A total of 21,018 articles were included, which in the recent two decades has increased by 125%. Among the top 12 hotspots, the annual productions rapidly increased in some topics, including *axonal regeneration signaling pathway*, *axon guidance cues*, *neural circuits and functional recovery*, *nerve conduits*, and *cells transplant*. Comparatively, the number of studies on *axon regeneration inhibitors* decreased. As for the topics focusing on *nerve graft and transplantation*, the annual number of papers tended to be relatively stable. Nevertheless, the underlying mechanisms of axon regrowth have not been completely uncovered. A lack of notable research on the epigenetic programs and noncoding RNAs regulation was observed. The significance of cell-type-specific data has been highlighted but with limited research working on that. Functional recovery from neuropathies also needs further studies.

**Conclusion:**

The last two decades witnessed tremendous progress in the field of axon regeneration. There are still a lot of challenges to be tackled in translating these technologies into clinical practice.

## Background

The nervous system is composed of two parts: the central nervous system (CNS—with the brain, the spinal cord, the optic and olfactory nerves) and the peripheral nervous system (PNS—comprises all the axons that innervate the whole body). While the PNS is able to regenerate, the CNS neurons are refractory to grow after injury [1]. Axons connect the nervous system with the rest of the body to ensure its proper function and the interaction with the outside world. However, any insult to the nervous system such as trauma, ischemia, neurodegeneration, and demyelination [2] leads to permanent impairment of motor, sensory, cognitive, and automatic nerve functions [3–5]. Thus, understanding mechanisms involved in nervous system repair is crucial not only for the neuroscience field but also for public health. Therefore, studies focusing on axon regeneration have become a research field with considerable interest.

For successful regeneration, the damaged axon needs to go through several critical steps: formation of growth cone-like structure, long-distance elongation, growth within the appropriate path, correct targeting to the proper partner, and functional circuits reconstruction. This fine and complicated journey is finely tuned by the expression of specific genes, the axon interactions with neighboring non-neuronal cells, and the extracellular environment [6]. Each phase is critical to achieve axon structural regeneration and functional recovery. Recently, the diversity of the neuronal populations has been highlighted as a key factor to take into consideration as they react differently to injury. It has been reported that within the retina, neurons forming the optic nerve, the retina ganglion cells (RGCs) show a different level of resilience and growth ability depending on their subtypes [7]. Axon injury triggers a sequence of molecular events, shifts in cellular organization, and responses depending on neuronal subtypes. The same pro-regenerative kinase, such as dual leucine sipper-bearing kinase (DLK), has been reported for promoting and accelerating axon regeneration in PNS [8], yet its activation alone could result in RGCs death [9], even if it is critical for regeneration as its deletion in a system that regenerates (upon mTOR activation for example) inhibits axon growth [9]. Decades of studies show that not only one molecular pathway but also several factors regulate axon regeneration [10]. However, so far, functional recovery remains still challenging as not all the regenerative pathways have been unlocked, and circuit’s formation is not achieved yet. More researches nowadays begin to focus on the multiple regulations to induce more robust axonal regrowth [10–12], and more scientists and engineers from different disciplines have combined their efforts together to make progress in this field [13, 14]. In order to overcome these puzzles and explore the optimal strategy for axonal regeneration, numerous institutions and worldwide researchers devote themselves to different parts of this research field.

Obtaining an overall perspective of the studies related to axonal regeneration is of vital importance to know the research hotspots and trends but also to assist in the design of multidimensional targeted strategies. As the quantitative analysis of academic literature, bibliometric analysis is commonly utilized on a given topic to evaluate the quantity of publishing literature, their academic impacts, research fields, or keywords [15]. It can efficiently elucidate the landscape of the existing publications, and it may also shed a light on the area requiring more effort.

Herein, we conducted a bibliometric analysis of the published literature on axon regeneration based on the database named Web of Science (WoS) over the past 22 years and applied an unsupervised machine learning named latent Dirichlet allocation (LDA) analysis to screen the sheer volume of literature. LDA is a generative probabilistic model to collect documents which has been widely used to uncover latent topics on a large scale [16, 17]. The results will address the research hotspots, publication trends, and the areas that lack evidences and might assist the researchers and the funding agencies to better know the areas of greatest research opportunities.

## Methods

### Data collection and general data analysis

To obtain eligible studies published from 2000 to 2021, our study conducted a literature search in the core collection of WoS, including Science Citation Index Expanded, Social Science Citation Index, Conference Proceeding Citation Index-Science, Conference Proceeding Citation index-Social Science and Humanities, Index Chemicus, and Current Chemical Reactions. The keywords used in search strategy were as follows: “axons,” “axon,” “axonal,” “regeneration,” “regrowth,” “reinnervation,” “repairment,” “repair,” and “recovery.” The general information of the search outcomes was extracted as an XLS file, including the titles, authors, affiliations, abstracts, publication years, total citations, journals, countries/regions, funding agencies, and research fields. The following bibliometric analysis was performed by using Microsoft Excel 2019 and Python version 3.7 based on the abstract of articles in the database. The associations between different countries/regions, funding agencies, or research fields were visualized by Gephi software version 0.9.2.

### Latent Dirichlet allocation (LDA) analysis

In the bibliometric analysis, a credible and good classification topic needs to identify a meaningful cluster of words [18]. Similar to the LDA, latent semantic indexing (LSI) and probabilistic latent semantic indexing (PLSI) are commonly used in topic modeling techniques either. However, Blei D. M. et al. [16] proposed that it is not clear for the reason to use LSI instead of more directly fitting models such as maximum likelihood or Bayesian methods. Besides, the PLSI usually has serious overfitting problems due to the linear growth of the number of parameters with the size of the corpus. To overcome such disadvantages, the LDA algorithm, a generative probabilistic model to collect documents, was proposed [16, 19]. The continuous data or other non-multinomial data can also easily be added to LDA. In the LDA algorithm, the “bag of words” technique is adopted to simplify each document into a vector of words, and then, the algorithm creates term vocabularies based on the frequency of words in the document [17]. Finally, the topics are characterized in terms of the probability distribution over words, and the documents are attributed to the topic based on probability. Before the implementation of LDA analysis, the number of topics needs to be prescribed, which is strongly related to the probability of topics being nonsensical [20]. Herein, the coherence value algorithm [20] is adopted to determine the optimal topic number instead of determining a topic number based on empiricism. The coherence value represents the arithmetic mean of all topic coherence. Each topic coherence represents the consistency of a topic which is measured by the semantic similarity between words vectors [21].

### Framework of the analysis

**Fig. 1:**
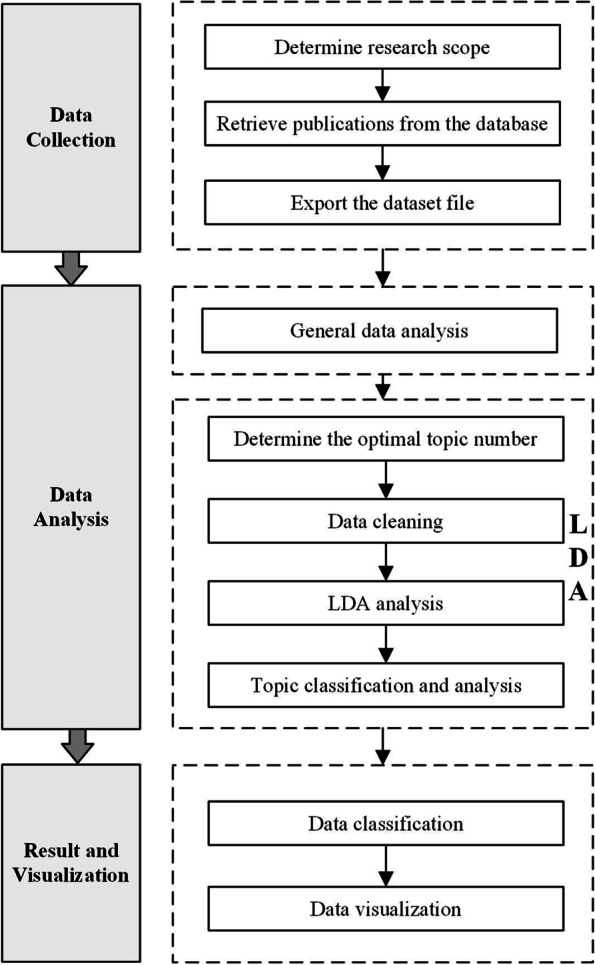
Workflow of the study. Schematic of the organization of the study into 3 parts: (1) data collection based on Web of Science, (2) data analysis using LDA method, and (3) results analysis and presentation

To illustrate the framework of the analysis procedure, the implementation steps are depictured in Fig. 1. The analysis is classified into three parts. First, the data collection is implemented based on the WoS to provide the database for the subsequent analyses. Second, general data analysis is conducted using Excel to investigate the distribution of publications, leading journals, leading countries/regions, funding agencies, and leading research fields. The LDA analysis is performed to identify different research topics and hotspots to obtain their relationships. Third, the visualizations of the results are implemented using Gephi version 0.9.2 and Origin version 9.1.

## Results

### Distribution of publications

**Fig. 2:**
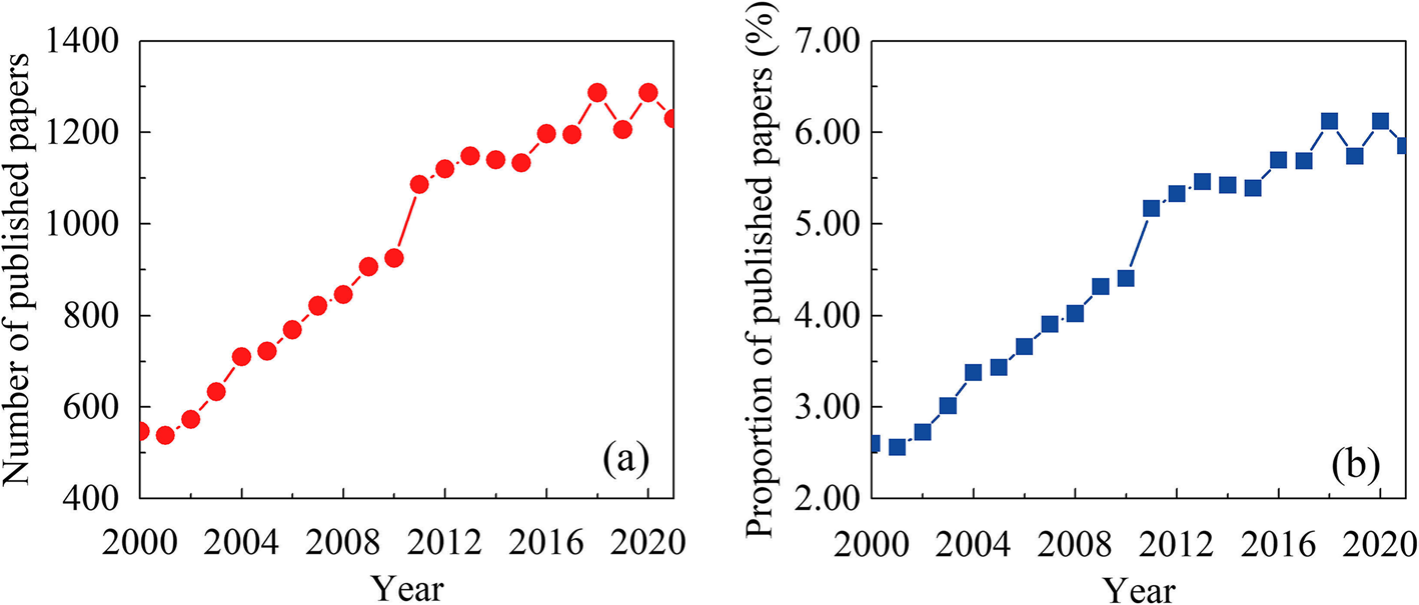
Publishing trend in axonal regeneration. The chronological distribution of the number (**a**) and proportion of published articles (**b**) related to axon regeneration

A total of 21,018 articles matching the retrieval criteria, as (“axons” or “axon” or “axonal”) and (“regeneration” or “regrowth” or “reinnervation” or “repairment” or “repair” or “recovery”), were identified. The chronological distribution of published articles from 2000 to 2021 is shown in Fig. 2. The publications were of sustained growth, and the number of articles rose sharply from 2010 (*n* = 925, 4.40%) to 2011 (*n* = 1086, 5.17%) and reached a peak in 2020 (*n* = 1287, 6.12%), and over half of enrolled articles (*n* = 13029; 61.99%) were published during the recent 10 years (2011–2021). To explore the evolution of the research focus over the years, Table 1 summarizes the ten most cited articles published in each decade. A closer look at the subject of the articles revealed that research interest has gradually shifted from the study of the inhibitory role of the environment of the injured axons, such as glial scar and myelin debris, to the essential role of neurons themselves, the functional recovery after injury, and the exploration of a beneficial role of astrocyte scar.

**Table 1:** Ten most cited articles published in each decade

Title	First author	Journal	Citations
Chondroitinase ABC promotes functional recovery after spinal cord injury	Elizabeth J. Bradbury (2002)	Nature	1813
Identification of two distinct macrophage subsets with divergent effects causing either neurotoxicity or regeneration in the injured mouse spinal cord	Kristina A. Kigerl (2009)	*Journal of Neuroscience*	1504
Demyelination increases radial diffusivity in corpus callosum of mouse brain	Sheng-Kwei Song (2005)	*Neuroimage*	1299
Nogo-A is a myelin-associated neurite outgrowth inhibitor and an antigen for monoclonal antibody IN-1	Maio S. Chen (2000)	*Nature*	1300
Reactivation of ocular dominance plasticity in the adult visual cortex	Tommaso Pizzorusso (2002)	*Science*	1165
Promoting axon regeneration in the adult CNS by modulation of the PTEN/mTOR pathway	Kevin Kyungsuk Park (2008)	*Science*	1091
Amyotrophic lateral sclerosis is a distal axonopathy: evidence in mice and man	Lindsey R. Fischer (2004)	*Experimental Neurology*	959
Identification of the No-go inhibitor of axon regeneration as a reticulon protein	Tadzia GrandPré (2000)	*Nature*	1011
Identification of a receptor mediating Nogo-66 inhibition of axonal regeneration	Alyson E. Fournier (2001)	*Nature*	971
Injection of adult neurospheres induces recovery in a chronic model of multiple sclerosis	Stefano Pluchino (2003)	*Nature*	901
Pancreatic cancer genomes reveal aberrations in axon guidance pathway genes	Andrew V. Biankin (2012)	*Nature*	1389
Astrocyte scar formation aids central nervous system axon regeneration	Mark A. Anderson (2016)	*Nature*	965
Systemic administration of exosomes released from mesenchymal stromal cells promotes functional recovery and neurovascular plasticity after stroke in rats	Hongqi Xin (2013)	*Journal of Cerebral Blood Flow and Metabolism*	598
Long-distance growth and connectivity of neural stem cells after severe spinal cord injury	Paul Lu (2012)	*Cell*	580
MiR-133b promotes neural plasticity and functional recovery after treatment of stroke with multipotent mesenchymal stromal cells in rats via transfer of exosome-enriched extracellular particles	Hongqi Xin (2013)	*Stem cells*	484
Sustained axon regeneration induced by co-deletion of PTEN and SOCS3	Fang Sun (2011)	*Nature*	479
Glial scar borders are formed by newly proliferated, elongated astrocytes that interact to corral inflammatory and fibrotic cells via STAT3-dependent mechanisms after spinal cord injury	Ina B. Wanner (2013)	*Journal of Neuroscience*	449
Macrophage-induced blood vessels guide Schwann cell-mediated regeneration of peripheral nerves	Anne-Laure Cattin (2015)	*Cell*	415
Microtubule stabilization reduces scarring and causes axon regeneration after spinal cord injury	Farida Hellal (2011)	*Science*	414
Grafted human-induced pluripotent stem-cell-derived neurospheres promote motor functional recovery after spinal cord injury in mice	Satoshi Nori (2011)	*Proceedings of the National Academy of Sciences of the United States of America*	375

### Leading journals

**Fig. 3:**
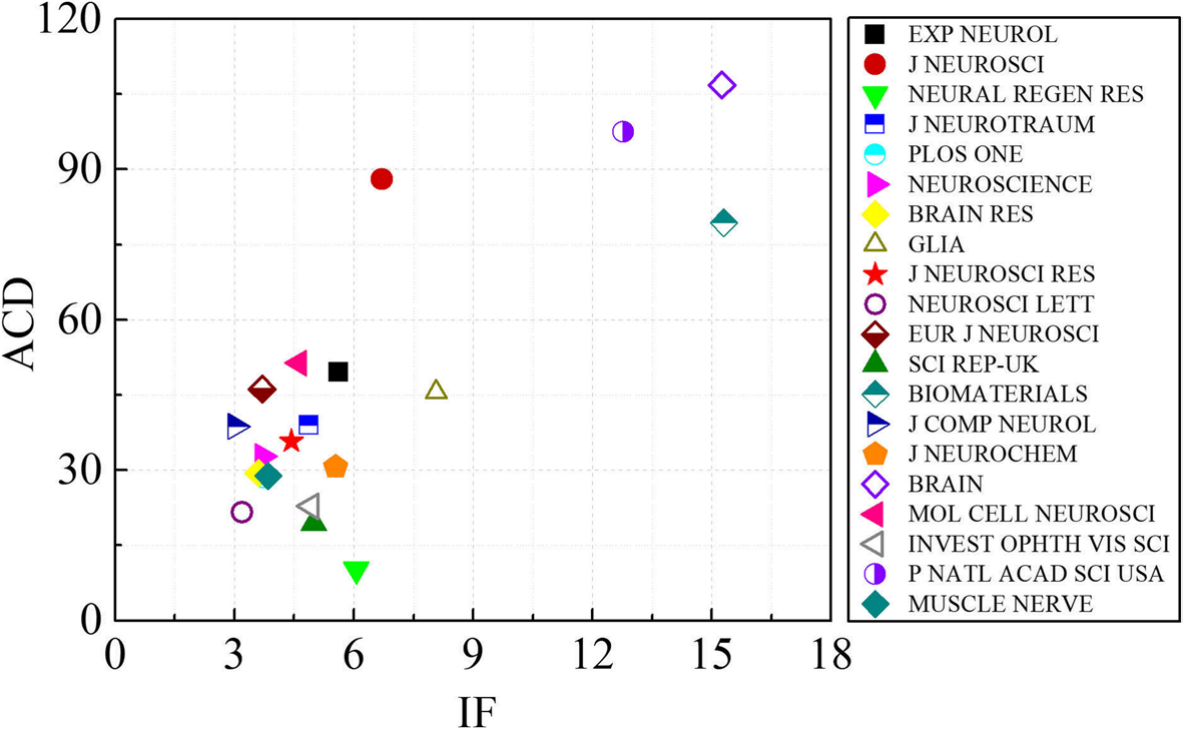
The impact evaluation of the leading journals. IF, impact factor; ACD the average number of citations per document. EXP NEUROL, *Experimental Neurology*; J NEUROSCI, *Journal of Neuroscience*; NEURAL REGEN RES, *Neural Regeneration Research*; J NEUROTRAUM, *Journal of Neurotrauma*; BRAIN RES, Brain Research, J NEUROSCI RES: Journal Of Neuroscience Research, NEUROSCI LETT: Neuroscience Letters, EUR J NEUROSCI: European Journal Of Neuroscience, SCI REP-UK: Scientific Reports, J COMP NEUROL: Journal Of Comparative Neurology, J NEUROCHEM: Journal Of Neurochemistry, MOL CELL NEUROSCI: Molecular And Cellular Neuroscience, INVEST OPHTH VIS SCI: Investigative Ophthalmology Visual Science, P NATL ACAD SCI USA: Proceedings Of The National Academy Of Sciences Of The United States Of America

Based on data analysis, the articles related to axonal regeneration from 2000 to 2021 were published in 2206 different journals. A third of these documents were published by the leading 20 journals listed in Table 2. The most prolific journal is *Experimental Neurology* with 826 publications in total, while the *Journal of Neuroscience* has the highest *H*-index. To identify the core journals in this research field, we further analyzed these journals by the combination of their impact factor (IF) and the average number of citations per document (ACD). As shown in Fig. 3, *Brain* is the journal possessing the largest ACD and IF simultaneously, followed by the *Proceedings of the National Academy of Sciences of the United States of America* and *Biomaterials*. As for the most influential journals, *Cell*, *Nature*, and *Science* published 13, 28, and 38 high-quality documents during this period, respectively.

**Table 2:** The top 20 journals in the field of axonal regeneration by TD

No.	Journal	TD	IF (2021)	TC	ACD	H-index
1	*Experimental Neurology*	826	5.62	40933	49.56	97
2	*Journal of Neuroscience*	708	6.709	62325	88.03	133
3	*Neural Regeneration Research*	544	6.058	5515	10.14	31
4	*Journal of Neurotrauma*	517	4.869	20138	38.95	74
5	*PLOS One*	430	3.752	12271	28.54	56
6	*Neuroscience*	334	3.708	10932	32.73	53
7	*Brain Research*	307	3.61	9041	29.45	48
8	*Glia*	289	8.073	13169	45.57	64
9	*Journal of Neuroscience Research*	268	4.433	9587	35.77	54
10	*Neuroscience Letters*	262	3.197	5656	21.59	37
11	*European Journal of Neuroscience*	257	3.698	11865	46.17	60
12	*Scientific Reports*	240	4.996	4648	19.37	38
13	*Biomaterials*	212	15.304	16826	79.37	78
14	*The Journal of Comparative Neurology*	212	3.028	8211	38.73	53
15	*Journal of Neurochemistry*	201	5.546	6160	30.65	48
16	*Brain*	192	15.255	20511	106.83	87
17	*Molecular and Cellular Neuroscience*	192	4.626	9861	51.36	58
18	*Investigative Ophthalmology & Visual Science*	178	4.925	4055	22.78	36
19	*Proceedings of the National Academy of Sciences of the United States of America*	170	12.779	16569	97.46	77
20	*Muscle and Nerve*	165	3.852	4759	28.84	35

*TD* the total number of documents, *IF* impact factor, *TC* total number of citations, *ACD* average number of citations per document

### Leading countries/regions and funding agencies

**Fig. 4:**
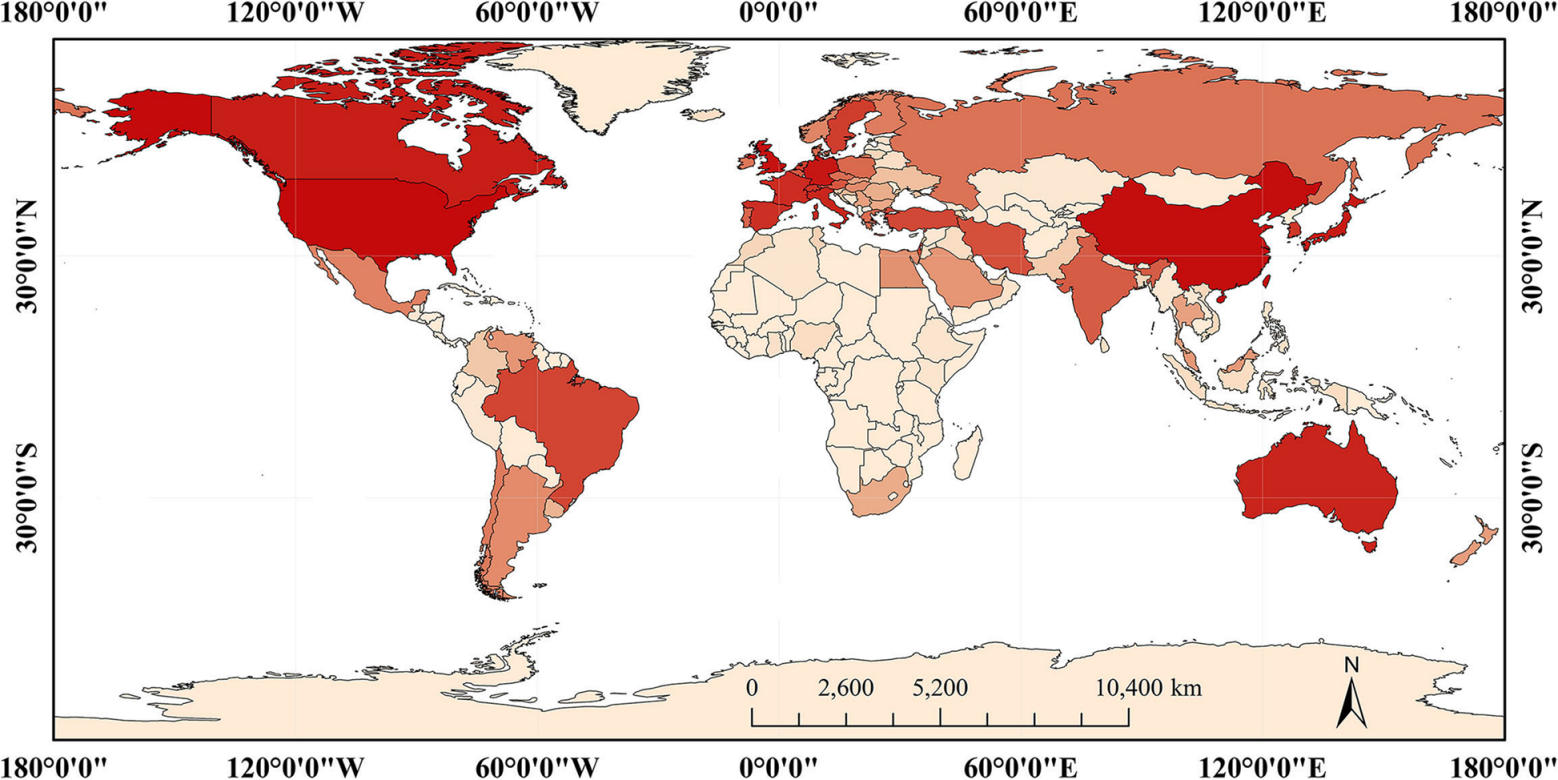
Geographical distributions of publications. Countries in a darker color indicate a higher ranking in the number of publications and vice versa. This schematic shows that the leading countries are the USA, P. R. China, and Germany

**Fig. 5:**
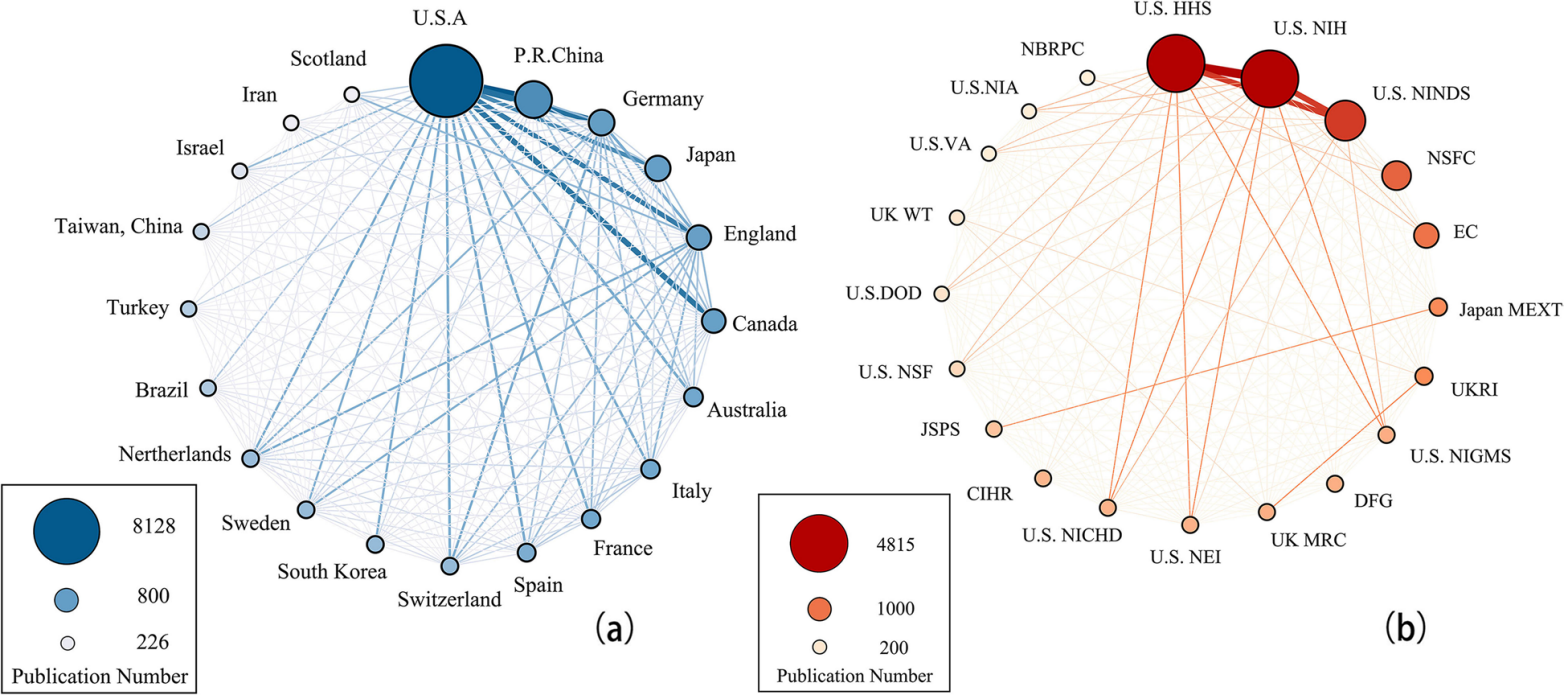
Global cooperation among the top 20 most productive countries/regions (**a**) and funding agencies (**b**). USA, the United States of America; P. R. China, People’s Republic of China; US HHS, United States Department of Health Human Services; US NIH, United States National Institutes Of Health; US NINDS, United States National Institute of Neurological Disorders Stroke; NSFC, National Natural Science Foundation of China; EC, European Commission; Japan MEXT, Japan Ministry of Education Culture Sports Science and Technology; UKRI, UK Research Innovation; US NIGMS, United States National Institute of General Medical Sciences; DFG, German Research Foundation; UK MRC, UK Medical Research Council; US NEI, United States National Eye Institute; US NICHD, United States Eunice Kennedy Shriver National Institute of Child Health Human Development; CIHR, Canadian Institutes of Health Research; JSPS, Japan Society for the Promotion of Science; US NSF, United States—National Science Foundation; US DOD, United States Department of Defense; UK WT, UK Wellcome Trust; US VA, United States Department Of Veterans Affairs; US NIA, United States National Institute on Aging; NBRPC, National Basic Research Program Of China

To gain insight into the worldwide academic contribution to the research on axon regeneration, we extracted the country/region and funding agencies of every article. Regarding the number of articles, all countries and regions are present in a different shade of colors (Fig. 4). The most productive country is the USA (8128, 38.67%) followed by China (3257, 15.50%) and Germany (1743, 8.29%). As for funding agencies, United States Department of Health Human Services is most prolific (4815, 22.90%), followed by United States National Institutes of Health (4780, 22.74%), United States National Institute of Neurological Disorders Stroke (2987, 14.21%), National Natural Science Foundation of China (1796, 8.55%), and European Commission (1346, 6.40%). Additionally, some researches were produced by multiple countries and regions, and the international cooperation was also analyzed by our study. It indicated that the researchers and organizations from the USA were extremely active in collaborative works with other countries and regions, especially with the top six prolific counties listed in Fig. 5a. However, in terms of funding agencies, the domestic cooperation dominates in different countries/regions (Fig. 5b).

### Leading research fields

**Fig. 6:**
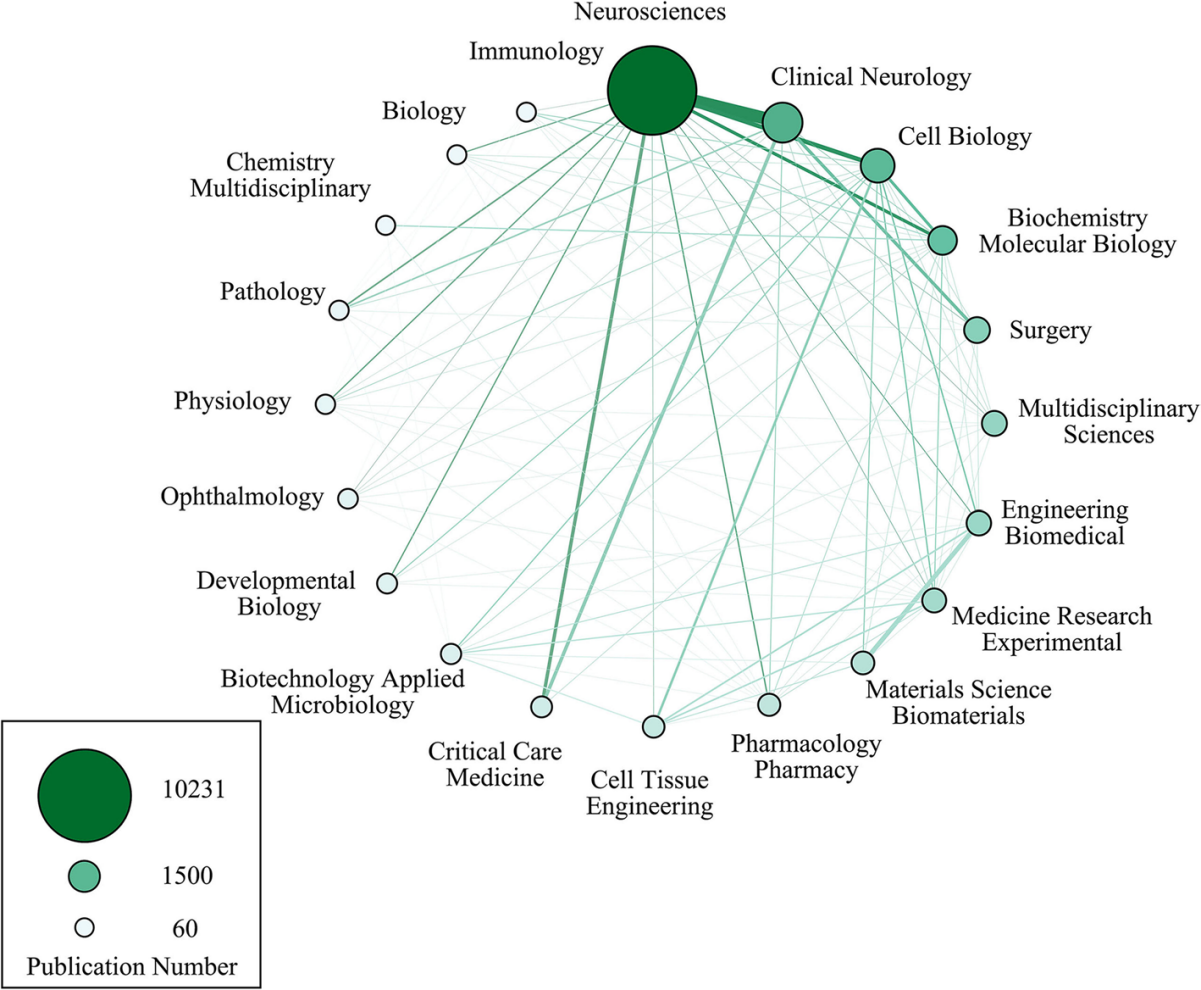
Cooperation among the top 20 most research field

With the increasing awareness of the significance and complexity of axon regeneration, cooperation of multiple disciplines has been achieved in this field. Our study extracted the information from the academic field per document and explored the association between these various disciplines. Undoubtedly, nearly half of the publications (10231, 48.68%) were published in the neurosciences field as illustrated in Fig. 6. Meanwhile, the work on axonal regeneration from clinical neurology, cell biology, biochemistry molecular and biology, surgery, multidisciplinary sciences, engineering biomedical, and medicine research experimental exceeded 1000 publications. Apart from the contribution from different disciplines mentioned above, the strongest cooperation was between neurosciences and clinical neurology. The research field of engineering biomedical was closely associated with that of material science biomaterials. In collaborative works, the involvement of cell biology, biochemistry molecular biology, critical care medicine, and cell tissue engineering could not be ignored, either.

### LDA analysis

**Fig. 7:**
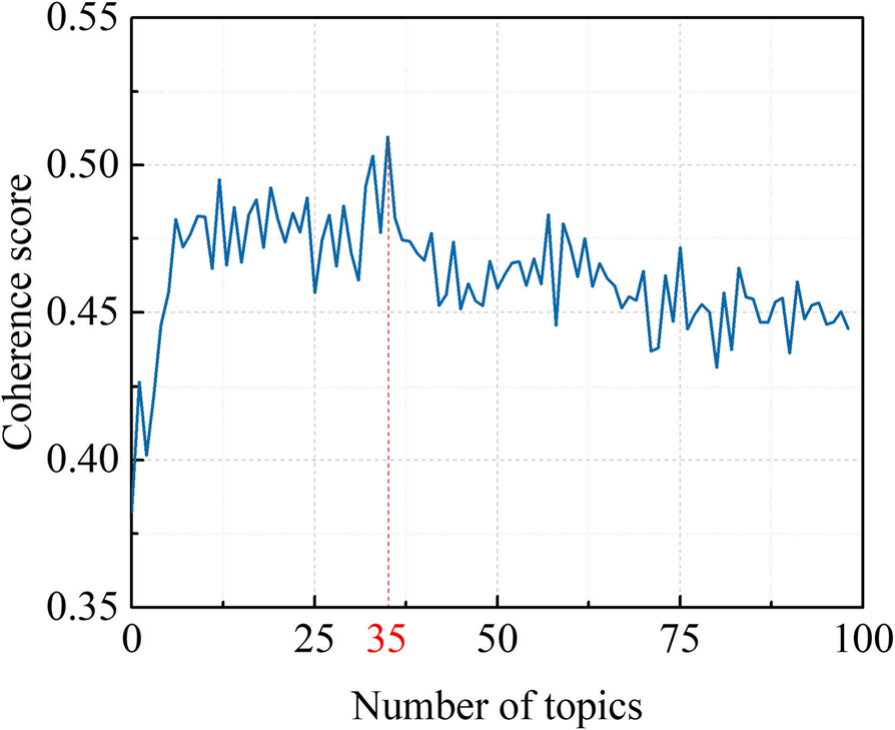
Coherence score for different numbers of topics

**Fig. 8:**
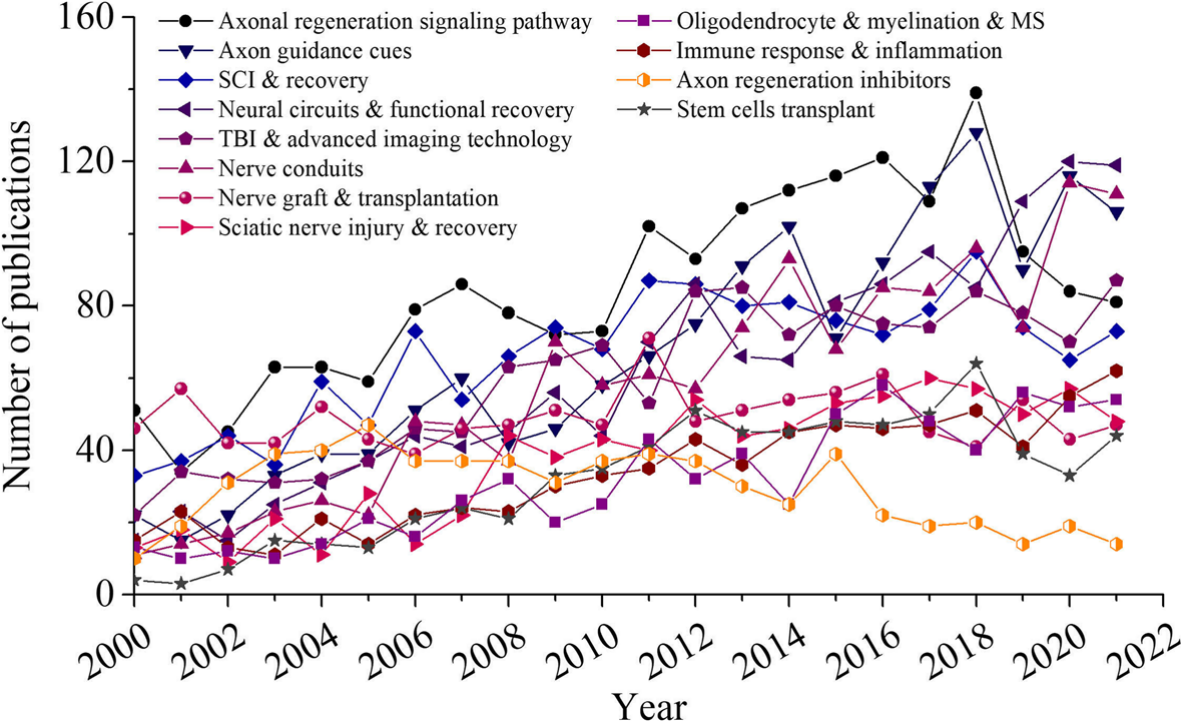
The trend of top 12 topics from 2000 to 2021. SCI, spinal cord injury; TBI, traumatic brain injury; MS, multiple sclerosis

To obtain a more specific perspective on the research hotspots, LDA was conducted to analyze abstracts from all documents to identify the topics that most frequently occur within the literature. After plenty of times data optimization by deleting the meaningless words such as “increased,” “significantly,” and “animal,” ten prominent feature terms, such as “astrocytes,” “transplantation,” and “nogo,” were extracted and generated into one topic after screening the content of each topic. According to Fig. 7, when the topic numbers increased to thirty-five, the calculation of coherence value reaches its maximum. Among these, the top 12 LDA-derived topics with the most publications were depicted in Fig. 8. Over the years, we observed the venue of new topics such as *axonal regeneration signaling pathway*, *axon guidance cues*, *neural circuits and functional recovery*, *nerve conduits*, *stem cells transplant, and oligodendrocyte, myelination, and multiple sclerosis (MS)*. In contrast, the number of studies related to *axon regeneration inhibitors* decreased during the same period. This observation strongly suggests a paradigm shift from the role of the extrinsic signals to neuronal intrinsic pathways involved in axon regeneration.

**Fig. 9:**
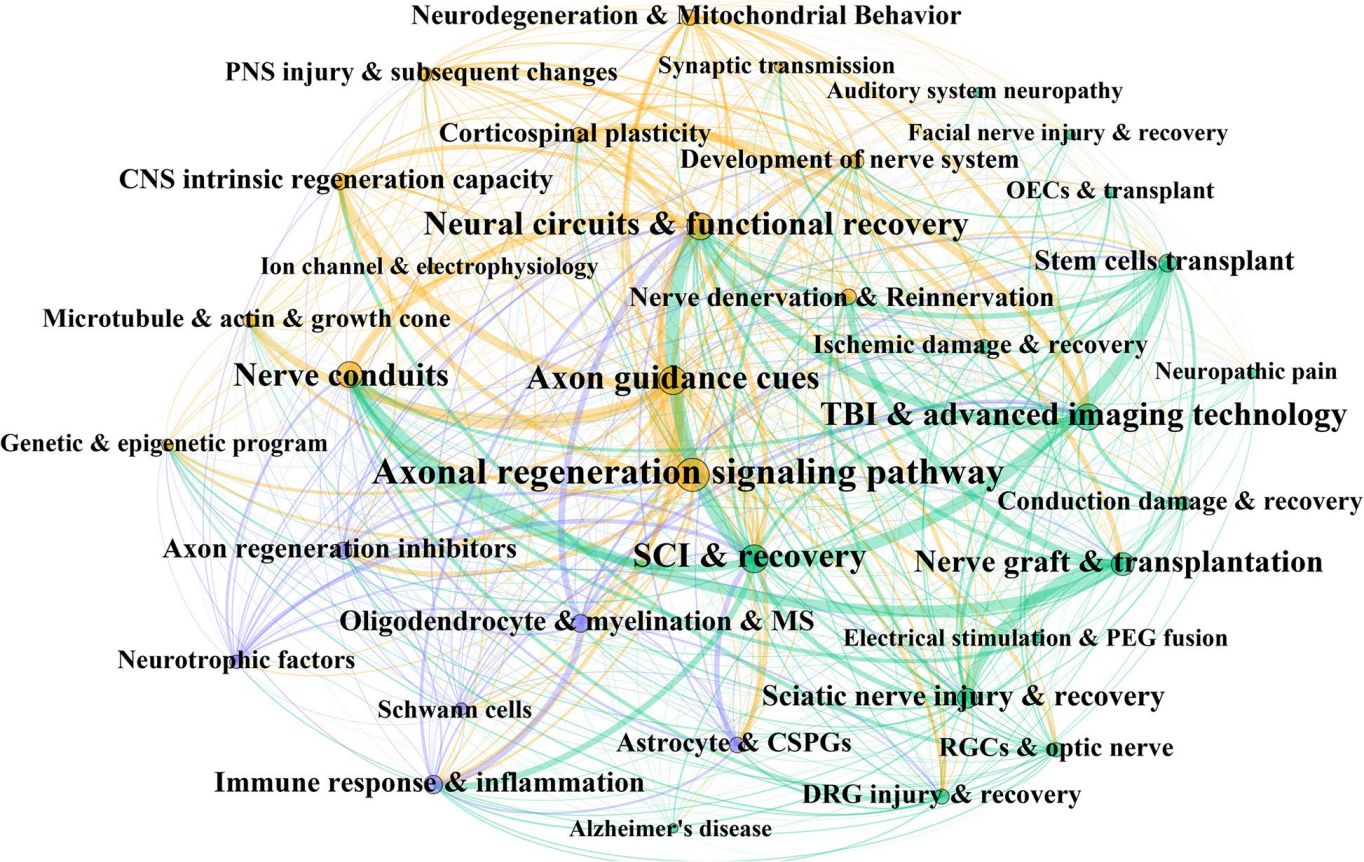
The network analysis of the research topics. The yellow cluster represents the “neuron intrinsic regeneration mechanisms,” the purple cluster represents the “non-neuron cells and factors” cluster, and the green cluster represents the “diseases/disease models and techniques” cluster

As for the topics focusing on *nerve graft and transplantation*, *spinal cord injury (SCI)*, *traumatic brain injury and imaging technology*, *sciatic nerve injury*, and *immune response and inflammation*, the annual production of papers tended to be relatively stable or slowly increasing. The network analysis of research topics is presented in Fig. 9, highlighting the topics of interest and their interrelationship. The number of publications per topic was represented by topic bubble size, while the magnitude of associations between topics was depicted by the network line width. Thirty-five LDA-derived topics were divided into 3 topic network clusters based on the Louvain method with respect to the degree of intrarelationship among publications and the content of articles. In the non-neuron cells and factor’s cluster, most topics were centered on neurotrophic factors, glia (through astrocytes, oligodendrocytes, Schwann cells, reactive gliosis, and myelination), and immune system (through immune cells and immune response). Within the cluster of neuron intrinsic regeneration mechanisms, in both CNS and PNS, every critical step of axon successful regeneration has been explored, including axonal regeneration signaling pathway, genetic and epigenetic program, axon guidance cues, microtubule and actin changes, growth cone formation, ion channel and electrophysiology, and mitochondrial behavior. The synaptic transmission, neuronal denervation and reinnervation, neural circuits, and functional recovery also featured prominently in this cluster. As for the cluster related to diseases/disease models and techniques, studies focusing on neuropathology in different body systems have been conducted, such as Alzheimer’s disease, MS, conduction damage, ischemic damage, auditory system neuropathy, and visual system neuropathy. Various neural injury models have been also intensively studied, including the retinal ganglion cell and explant, sciatic nerve injury model, dorsal root ganglion, and SCI model. Some cutting-edge therapies have shown up, and plenty of treatments remained in a continuously optimizing process, such as nerve conduit, cell-based therapy, and nerve graft transplantation.

## Discussion

The last two decades witnessed tremendous progress in the field of axon regeneration. These breakthroughs go with amazing technological evolution allowing a better understanding of the biology of the nervous system. Nevertheless, the underlying mechanisms of axon regrowth have not been completely uncovered as functional recovery remains challenging. People suffering from nervous system insults still have to deal with permanent motor, sensory, cognitive impairments, and automatic nervous system dysfunction. Thus, understanding axon regeneration is not only the key for neuroscience but also is a major clinically relevant problem and a critical public health issue. In our study, we have revealed how the field of axon regeneration is progressing in terms of number of publications, hotspots, scientific fields, the countries/regions, and funding agencies involved. The following systematic review mainly focuses on the trend and top hotspots in this research field.

### Role of neuronal intrinsic pathways to CNS regeneration

According to our results, a significant increase in the number of publications is observed between 2010 and 2011, especially the publications related to *axonal regeneration signaling pathway*. Over the 22 years, *axon regeneration inhibitors* are the only hotspots showing a decreased rate in annual production. It is highly likely that this progression is correlated with a paradigm shift in the field of axon regeneration. Since the early studies on axon regeneration from David and Aguayo [22], it has been admitted that the environment of the mature CNS is refractory for regeneration [23, 24]. This study led to identify the role of myelin debris, the players of the glial scar and the inhibitory factors secreted by these cells such as the chondroitin sulfate proteoglycans, and the neuroinflammatory response [24–26]. The corresponding receptors and potential intracellular signaling pathways were also subsequently explored, such as Nogo receptors and its co-receptors, protein tyrosine phosphatases σ, RhoA/Rho kinase, and Akt/GSK-3β signaling pathways [27, 28]. However, the inhibition of these pathways in vivo only induced limited regeneration [29]. These results implied that other pathways or mechanisms might be involved in mature CNS regeneration. Interestingly, developing neurons have tremendous growth capability that declines within the development process [30]. Zhigang He’s group explored the idea of activating growth-related molecular pathways within mature neurons to restore neuronal growth capability. By using the optic nerve as model, Park et al. showed for the first that neuronal activation of mTOR pathway leads to robust regeneration and neuroprotection. In 2010, it has been shown that mTOR activation in cortical spinal neurons induces spinal cord regeneration [31]. Intact axons from adult mammalian CNS show considerable capacity for structural plasticity and collateral sprouting [32, 33]. However, upon injury, CNS axons are not able to regenerate. Thus, it might suggest that the impaired intrinsic regenerative capability was also attributed to the injury and the downstream stress responses [10]. Consistent with the intrinsic capability of regeneration of neurons, a large number of studies were conducted, and many novel targets have been found, such as injury-induced signals and ion channel changes, growth cone formation and regulation, long-distance axonal extension and guidance, axonal transport, and soma reactions [23]. These studies allowed to improve the understanding of regenerative-competent neuron mechanisms from injury signaling pathways (both rapid phase and delayed phase) to genetic and transcriptional changes [34]. Some studies also provided strategies to regenerate injured axons by modulating different signaling pathways such as the melanopsin/GPCR (G protein-coupled receptor) [35], the PTEN (phosphatase and tensin homolog)/mTOR (mammalian target of rapamycin) [30] and the cAMP/PKA (protein kinase A) and PI3K (phosphatidylinositol 3-kinase) [36].

### Axon regeneration is a multifactor process

With the in-depth research in both the extrinsic environment and intrinsic competence of regrowth, another notion becomes more and more clear that no single molecule or signaling pathway could be responsible for successfully and precisely axonal regeneration in all neuronal types. It has been shown, for example, that the combination of protein translation and activation of gene transcription induces sustained axon regeneration [10, 11, 37]. The activation of these pathways has a synergistic effect on axon regrowth. de Lima et al. (2012) [38] used the activation of protein translation with increased inflammation and the activation of intracellular messenger such as cAMP. By this mean, axons are able to grow from the eyeballs to the brain part of the visual system. The proteomics analysis of the specific neuronal response to axon injury revealed that several molecular pathways are involved. By modulating some of these pathways, Belin et al. (2015) [10] show that extensive axon regeneration could be obtained with many axons reaching the optic chiasm and even into the brain. Neuronal activity is key to building functional circuits [39]. Thus, Lim et al. (2016) [40] combined the activated mTOR pathway with neuronal activity for sufficient elongation and the inhibition of neuronal activity by using a designer receptor exclusively activated by a designer drug (DREADS) compromises axon growth. From all these studies, it is clear that using combinatorial approaches could be a novel research direction in the future.

### The rapid rise of guidance cues topic

The topics *axon guidance cues* and *neural circuits and functional recovery* also belonged to hotspots implying another research direction with increasing efforts. For successful axonal regeneration, except for regaining the capability of regrowth, the final purpose is to navigate properly, achieve neural circuits reconstruction and completely functional recovery. By the end of the twentieth century, the four prominent axon guidance families have been discovered, including ephrins, semaphorins, netrins, and slits. The sequent studies have revealed the detailed structure and underlying function of all members from the four major families. For instance, ephrins consist of two structurally distinct proteins (ephrin A and ephrin B), which could interact with different Eph receptors and further trigger different signaling pathways [41, 42]. Netrin-1 combined with different receptors (DCC or Unc5 proteins) can induce axon chemoattraction or repulsion, respectively [43]. Other guidance molecules and receptors also have been identified, such as Sonic hedgehog, Wnt, and bone morphogenetic protein (BMP) [44–46]. However, compared to the huge number of axons, different types of neural populations, and the complexity of neural circuits formation, the number of the existing guidance cues is pretty small. An emerging theme that axon guidance receptors interact with not only ligands but also receptors or coreceptors has developed [47]. In this way, the limited guidance cues could produce diversified signaling outcomes. Additionally, the plasticity response of the growth cone is another way to solve the disparity between relatively few guidance cues and sophisticated neural wiring. Under the modulation by the intrinsic and extrinsic environment, the growth cone regulates spatiotemporal activation of receptors to induce corresponding downstream signaling events, and the growth cone and cytoskeleton can be rearranged to achieve the targeted guidance [48]. Nevertheless, the mechanism of precisely spatial and temporal regulation of different effect receptors in different cells still needs more studies.

### Nerve grafts and conduits are hot topics

*Nerve graft and transplantation* is the only hotspots with a steady rate of annual production. It is crucial to the nerve gap that cannot be surgically coaptated without tension. From the period of World War I [49], autologous nerve transplantation (ANT) is the clinical gold standard for bridging a nerve gap, as advantages of ANT include the non-immunogenicity and the reconstruction for large nerve gaps [50]. The consequent researches have been aroused with respect to the clinical technique of surgeries, the range of application of specific donor nerve, and the graft vascularization [51, 52]. However, the finite number of autologous donor nerves, the comorbidities of the donor site, and the complications at the operation site could result in imperfectly structural and functional recovery, even with unwanted issues such as sensibility decrease, paresthesia, and neuronal pain. These challenges provide fertile ground for the development of a wide range of advanced biomaterials and synthetic materials as alternatives to the ANT [53]. It is the reason that the topic *nerve graft and transplantation* was closely associated with *nerve conduits*, which was demonstrated by the network analysis. The nerve conduits were developed and updated to mimic native nerves with tailored mechanical and physical scaffolds, cellular components, and molecular signals that accelerate axon regrowth and adjust the immune system [54]. Nevertheless, the conduits of degradation rate, swelling, and biocompatibility have still plagued researchers [55]. Besides, the property that the interaction of native nerve with the surrounding tissues also needs more studies to work out. Incorporating multiple factors might be a new direction of the conduits to mimic native nerve better.

### Hotspots in disease and disease models

Thus far, we have understood the significance of axon regrowth, elongation, guidance, and circuit’s formation. The faulty of any step may lead to irreversible neuropathy. As a severe and devastating disease, SCI is one of the top three hotspots in the current study. This disease can lead to numerous types of motor, sensory dysfunction, and neurological disorders, such as loss or reduction in urinary, intestinal, and sexual functions, but it still lacks effective treatments in clinical practice. With the advent of stem cell technology, different stem cells have been explored for treating SCI. Recently, the phases 1–2 clinical trials of umbilical cord blood mononuclear cell transplant therapy have shown this transplantation safety and the function recovery of motor, bowel, and bladder in patients suffering from chronic complete SCI [56]. In the Puerta de Hierro phase 1/2 clinical trials, applying autologous bone marrow adult mesenchymal stem cells by intrathecal injection, all SCI patients including people with the longest disease duration have gradually recovered in clinical parameters [57]. The application of induced pluripotent stem cell (iPSC) is another promising technology with less ethical problems and higher self-renewal ability [58]. iPSCs have been proven the capability of promoting axon regeneration and myelination and improving the local environment in SCI animal models [59, 60]. Currently, the first clinical study of transplanting iPSC-derived cells to SCI patients has been launched by the Okano Laboratory group [61, 62]. In pursuit of meaningful functional recovery in SCI, recently, combinatorial treatments containing biochemical molecules, biomaterials, cell-based therapy, and rehabilitation exercises have also emerged and rapidly developed [63, 64].

Given the complexity of human systems and clinical trials, the development of experimental animal models or in vitro systems to address nerve defects is highlighted [23]. For instance, SCI models are commonly used for studies focusing on injured neurons in CNS. As for the research on PNS, the sciatic nerve injury model and dorsal root ganglions were frequently employed. Comparatively, the studies with retinal ganglion cells or retinal explants which is an ideal model to study population specific regenerative response or long-distance axon regeneration are less used. It might partly have to do with the difficulties of the sophisticated manipulation with the micro-instruments and the comprehensive understanding of the visual system. To explore cellular and molecular events of axon injury and degeneration at the single-axon level, the laser axotomy, axonal stretching and microfluidic compartmentalized neuronal culture models, and other in vitro models have been developed [65]. It is notable that microfluidic chamber systems enable the spatial isolation between axons and somas to study the specific subcellular areas [66]. Moreover, this system can be modified and updated for different purposes, such as the 3D co-culture between CNS neurons and glia in a vertically layered platform [67].

### Topics that need further research

Our analysis identified a lack of notable research on epigenetic programs and noncoding RNAs regulation which is of vital significance at the posttranscriptional level. Few studies have elucidated the roles of some noncoding RNAs, such as the effect of microRNAs (miR-20a and miR-128) in promoting neurite extension [68]. Only several researches were aimed to find out the epigenetic mechanism regulating axonal regeneration, such as the histone acetyltransferase p300/CBP-associated factor (PCAF)-dependent epigenetic changes [69] and ubiquitin-like containing PHD ring finger 1 (UHRF1)-dependent DNA methylation to promote axon regeneration [70]. However, numerous unknown alterations need to be discovered, and many challenges remain. Progressive modern technology could be the opportunity to allow in-depth exploitation, such as the combination of omics from different molecular levels (transcriptomic, proteomic, and epigenomic). Benefiting from the advances in experimental methodology, such as single-cell RNA sequencing, the heterogeneity of transcriptomic responses to injury, selective gene expression, and physiological and morphological changes have been observed in different neuronal populations [12, 71]. Hence, the significance of cell-type-specific data has been highlighted but with limited researches working on that. There are still a lot of challenges to be tackled in translating these technologies into clinical practice. The functional recovery from neuropathies in other systems also warrants further studies, such as the visual system, auditory system, and urinary system, which also emphasizes the significance of multidisciplinary cooperation in future researches.

### Limitation

Admittedly, there were some limitations of the current study. The literature search was only on the basis of the WOS collection indexed journals, which is a common limitation of similar studies [72, 73]. Future bibliometric studies could be conducted on other medical science databases, which would offer a supplement to more comprehensively understand this field. Despite the LDA analysis can extract not only the topic of articles more precisely and easily but also the connections between different topics can be analyzed, the LDA theme was created by artificial intelligence, and these themes were on the basis of machine-driven understanding. Moreover, small specific niches were not spotted by this analysis. Indeed, in this study, we did not highlight key studies in the field of CNS repair such as the work from the Courtine lab; they combine pharmacological approach to engineering to activate dormant circuits in order to allow functional recovery [74–76]. Hence, we combined a manual and detailed review and exploration of these topics to deliver a deeper understanding of the trends, hotspots, and even the gaps in the research field of axonal regeneration.

## Conclusions

This study is the first bibliometric analysis that assessed the trend and hotspots in the research field of axonal regeneration based on publications over 22 years. An overall perspective of axonal regeneration assists not only in understanding the existing achievements, the core published journals, and the cooperation between countries/regions, funding agencies, or research fields but also in discovering the research gaps and creating possibilities for multidisciplinary and multidimensional consociation to work out the mystery of axonal regrowth together.

## Data Availability

The data that support the findings of this study are available from the corresponding author upon reasonable request.

## Abbreviations

CNS: Central nervous system
PNS: Peripheral nervous system
RGC: Retina ganglion cells
DLK: Dual leucine zipper-bearing kinase
WoS: Web of Science
LDA: Latent Dirichlet Allocation
IF: Impact factor
ACD: Average number of citations per document
MS: Multiple sclerosis
SCI: Spinal cord injury
GPCR: G protein-coupled receptor
PTEN: Phosphatase and tensin homolog
mTOR: Mammalian target of rapamycin
PKA: Protein kinase A
iPSC: Induced pluripotent stem cell
PI3K: Phosphatidylinositol 3-kinase
DREADS: Designer receptor exclusively activated by a designer drug
ANT: Autologous nerve transplantation
PCAF: p300/CBP-associated factor
UHRF1: Ubiquitin-like containing PHD ring finger 1

## References

[1] Fawcett JW (2020). The struggle to make CNS axons regenerate: why has it been so difficult?. Neurochem Res.

[2] Muzio MR, Cascella M (2022). Histology, axon.

[3] Compston A, Coles A (2008). Multiple sclerosis. Lancet..

[4] Mishra AK, Dixit A (2021). Dopaminergic axons: key recitalists in Parkinson’s disease. Neurochem Res.

[5] Sharma K, Amin MAM, Gupta N, Zinman L, Zhou X, Irving H (2020). Retinal spheroids and axon pathology identified in amyotrophic lateral sclerosis. Invest Ophthalmol Vis Sci.

[6] Raivich G, Makwana M (2007). The making of successful axonal regeneration: genes, molecules and signal transduction pathways. Brain Res Rev.

[7] Duan X, Qiao M, Bei F, Kim IJ, He Z, Sanes JR (2015). Subtype-specific regeneration of retinal ganglion cells following axotomy: effects of osteopontin and mtor signaling. Neuron..

[8] Shin JE, Cho Y, Beirowski B, Milbrandt J, Cavalli V, DiAntonio A (2012). Dual leucine zipper kinase is required for retrograde injury signaling and axonal regeneration. Neuron..

[9] Watkins TA, Wang B, Huntwork-Rodriguez S, Yang J, Jiang Z, Eastham-Anderson J (2013). Dlk initiates a transcriptional program that couples apoptotic and regenerative responses to axonal injury. Proc Natl Acad Sci U S A.

[10] Belin S, Nawabi H, Wang C, Tang S, Latremoliere A, Warren P (2015). Injury-induced decline of intrinsic regenerative ability revealed by quantitative proteomics. Neuron..

[11] Sun F, Park KK, Belin S, Wang D, Lu T, Chen G (2011). Sustained axon regeneration induced by co-deletion of Pten and Socs3. Nature..

[12] Tran NM, Shekhar K, Whitney IE, Jacobi A, Benhar I, Hong G (2019). Single-cell profiles of retinal ganglion cells differing in resilience to injury reveal neuroprotective genes. Neuron..

[13] Stewart CE, Kan C, Stewart BR, Sanicola HR, Jung JP, Sulaiman O (2020). Machine intelligence for nerve conduit design and production. J Biol Eng.

[14] Li R, Li DH, Zhang HY, Wang J, Li XK, Xiao J (2020). Growth factors-based therapeutic strategies and their underlying signaling mechanisms for peripheral nerve regeneration. Acta Pharmacol Sin.

[15] Wei WJ, Shi B, Guan X, Ma JY, Wang YC, Liu J (2019). Mapping theme trends and knowledge structures for human neural stem cells: a quantitative and co-word biclustering analysis for the 2013-2018 period. Neural Regen Res.

[16] Blei DM, Ng AY, Jordan MI (2003). Latent Dirichlet allocation. J Mach Learn Res.

[17] Stout NL, Alfano CM, Belter CW, Nitkin R, Cernich A, Lohmann SK (2018). A bibliometric analysis of the landscape of Cancer Rehabilitation Research (1992-2016). J Natl Cancer Inst.

[18] Bhat MR, Kundroo MA, Tarray TA, Agarwal B (2020). Deep LDA : a new way to topic model. J Inf Optim Sci.

[19] Syed S, Spruit M. Full-Text Or Abstract? Examining Topic Coherence Scores Using Latent Dirichlet Allocation. In: IEEE International Conference on Data Science and Advanced Analytics (DSAA). Tokyo: Institute of Electrical and Electronics Engineers; 2017. paper no. 165–74. 10.1109/DSAA.2017.61.

[20] Mimno D, Wallach H, Talley E, Leenders M, McCallum A. Optimizing Semantic Coherence in Topic Models. In: Proceedings of the 2011 Conference on Empirical Methods in Natural Language Processing (EMNLP). Edinburgh: Association for Computational Linguistics; paper no. 262–72. https://aclanthology.org/D11-1024.

[21] Liu Q, Zheng Z, Zheng J, Chen Q, Liu G, Chen S (2020). Health communication through news media during the early stage of the covid-19 outbreak in China: digital topic modeling approach. J Med Internet Res.

[22] David S, Aguayo AJ (1981). Axonal elongation into peripheral nervous system "ridges" after central nervous system injury in adult rats. Science..

[23] He Z, Jin Y (2016). Intrinsic control of axon regeneration. Neuron..

[24] Yiu G, He Z (2006). Glial inhibition of CNS axon regeneration. Nat Rev Neurosci.

[25] Geoffroy CG, Zheng B (2014). Myelin-associated inhibitors in axonal growth after CNS injury. Curr Opin Neurobiol.

[26] Donnelly DJ, Popovich PG (2008). Inflammation and its role in neuroprotection, axonal regeneration and functional recovery after spinal cord injury. Exp Neurol.

[27] Sami A, Selzer ME, Li S (2020). Advances in the signaling pathways downstream of glial-scar axon growth inhibitors. Front Cell Neurosci.

[28] Boghdadi AG, Teo L, Bourne JA (2018). The involvement of the myelin-associated inhibitors and their receptors in CNS plasticity and injury. Mol Neurobiol.

[29] Lee JK, Geoffroy CG, Chan AF, Tolentino KE, Crawford MJ, Leal MA (2010). Assessing spinal axon regeneration and sprouting in Nogo-, Mag-, And Omgp-deficient mice. Neuron.

[30] Park KK, Liu K, Hu Y, Smith PD, Wang C, Cai B (2008). Promoting axon regeneration in the adult CNS by modulation of the Pten/Mtor pathway. Science..

[31] Liu K, Lu Y, Lee JK, Samara R, Willenberg R, Sears-Kraxberger I (2010). Pten deletion enhances the regenerative ability of adult corticospinal neurons. Nat Neurosci.

[32] Rosenzweig ES, Courtine G, Jindrich DL, Brock JH, Ferguson AR, Strand SC (2010). Extensive spontaneous plasticity of corticospinal projections after primate spinal cord injury. Nat Neurosci.

[33] Holtmaat A, Svoboda K (2009). Experience-dependent structural synaptic plasticity in the mammalian brain. Nat Rev Neurosci.

[34] Mahar M, Cavalli V (2018). intrinsic mechanisms of neuronal axon regeneration. Nat Rev Neurosci.

[35] Li S, Yang C, Zhang L, Gao X, Wang X, Liu W (2016). Promoting axon regeneration in the adult Cns by modulation of the melanopsin/Gpcr signaling. Proc Natl Acad Sci U S A.

[36] Ohtake Y, Sami A, Jiang X, Horiuchi M, Slattery K, Ma L (2019). Promoting axon regeneration in adult CNS by targeting liver kinase B1. Mol Ther.

[37] Chen L, Chuang M, Koorman T, Boxem M, Jin Y, Chisholm AD (2015). Axon injury triggers Efa-6 mediated destabilization of axonal microtubules via Tacc and doublecortin like kinase. Elife..

[38] de Lima S, Koriyama Y, Kurimoto T, Oliveira JT, Yin Y, Li Y (2012). Full-length axon regeneration in the adult mouse optic nerve and partial recovery of simple visual behaviors. Proc Natl Acad Sci U S A.

[39] Schafer DP, Lehrman EK, Kautzman AG, Koyama R, Mardinly AR, Yamasaki R (2012). Microglia sculpt postnatal neural circuits in an activity and complement-dependent manner. Neuron..

[40] Lim JH, Stafford BK, Nguyen PL, Lien BV, Wang C, Zukor K (2016). Neural activity promotes long-distance, target-specific regeneration of adult retinal axons. Nat Neurosci.

[41] Pasquale EB (2008). Eph-Ephrin bidirectional signaling in physiology and disease. Cell..

[42] Seiradake E, Harlos K, Sutton G, Aricescu AR, Jones EY (2010). An extracellular steric seeding mechanism for Eph-Ephrin signaling platform assembly. Nat Struct Mol Biol.

[43] Xu K, Wu Z, Renier N, Antipenko A, Tzvetkova-Robev D, Xu Y (2014). Neural migration. Structures of Netrin-1 bound to two receptors provide insight into its axon guidance mechanism. SCIENCE..

[44] Charron F, Stein E, Jeong J, McMahon AP, Tessier-Lavigne M (2003). The morphogen Sonic hedgehog is an axonal chemoattractant that collaborates with Netrin-1 in midline axon guidance. Cell..

[45] Lyuksyutova AI, Lu CC, Milanesio N, King LA, Guo N, Wang Y (2003). Anterior-posterior guidance of commissural axons by Wnt-Frizzled signaling. Science..

[46] Chédotal A (2019). Roles of axon guidance molecules in neuronal wiring in the developing spinal cord. Nat Rev Neurosci.

[47] Zang Y, Chaudhari K, Bashaw GJ (2021). New insights into the molecular mechanisms of axon guidance receptor regulation and signaling. Curr Top Dev Biol.

[48] Dickson BJ (2002). Molecular mechanisms of axon guidance. Science..

[49] Naff NJ, Ecklund JM (2001). History of peripheral nerve surgery techniques. Neurosurg Clin N Am.

[50] Socolovsky M, Di Masi G, Battaglia D (2011). Use of long autologous nerve grafts in brachial plexus reconstruction: factors that affect the outcome. Acta Neurochir.

[51] Broeren BO, Duraku LS, Hundepool CA, Walbeehm ET, Zuidam JM, Hooijmans CR (2021). Nerve recovery from treatment with a vascularized nerve graft compared to an autologous non-vascularized nerve graft in animal models: a systematic review and meta-analysis. PLoS One.

[52] Ruijs AC, Jaquet JB, Kalmijn S, Giele H, Hovius SE (2005). Median and ulnar nerve injuries: a meta-analysis of predictors of motor and sensory recovery after modern microsurgical nerve repair. Plast Reconstr Surg.

[53] Gao Y, Wang YL, Kong D, Qu B, Su XJ, Li H (2015). Nerve autografts and tissue-engineered materials for the repair of peripheral nerve injuries: a 5-year bibliometric analysis. Neural Regen Res.

[54] Meena P, Kakkar A, Kumar M, Khatri N, Nagar RK, Singh A (2021). Advances and clinical challenges for translating nerve conduit technology from bench to bed side for peripheral nerve repair. Cell Tissue Res.

[55] Nectow AR, Marra KG, Kaplan DL (2012). Biomaterials for the development of peripheral nerve guidance conduits. Tissue Eng Part B Rev.

[56] Zhu H, Poon W, Liu Y, Leung GK, Wong Y, Feng Y (2016). Phase I-Ii clinical trial assessing safety and efficacy of umbilical cord blood mononuclear cell transplant therapy of chronic complete spinal cord injury. Cell Transplant.

[57] Vaquero J, Zurita M, Rico MA, Bonilla C, Aguayo C, Montilla J (2016). An approach to personalized cell therapy in chronic complete paraplegia: the Puerta de Hierro phase I/Ii clinical trial. Cytotherapy..

[58] Martin-Lopez M, Fernandez-Muñoz B, Canovas S (2021). Pluripotent stem cells for spinal cord injury repair. Cells Basel.

[59] Fan L, Liu C, Chen X, Zou Y, Zhou Z, Lin C (2018). Directing induced pluripotent stem cell derived neural stem cell fate with a three-dimensional biomimetic hydrogel for spinal cord injury repair. ACS Appl Mater Interfaces.

[60] Lu P, Woodruff G, Wang Y, Graham L, Hunt M, Wu D (2014). Long-distance axonal growth from human induced pluripotent stem cells after spinal cord injury. Neuron..

[61] Tsuji O, Sugai K, Yamaguchi R, Tashiro S, Nagoshi N, Kohyama J (2019). Concise review: laying the groundwork for a first-in-human study of an induced pluripotent stem cell-based intervention for spinal cord injury. Stem Cells.

[62] Sugai K, Sumida M, Shofuda T, Yamaguchi R, Tamura T, Kohzuki T (2021). First-in-human clinical trial of transplantation of Ipsc-derived Ns/Pcs in subacute complete spinal cord injury: study protocol. Regen Ther.

[63] Yang B, Zhang F, Cheng F, Ying L, Wang C, Shi K (2020). Strategies and prospects of effective neural circuits reconstruction after spinal cord injury. Cell Death Dis.

[64] Dalamagkas K, Tsintou M, Seifalian A, Seifalian AM. Translational regenerative therapies for chronic spinal cord injury. Int J Mol Sci. 2018;19(6). 10.3390/ijms19061776. 10.3390/ijms19061776 PMC6032191 29914060

[65] Varier P, Raju G, Madhusudanan P, Jerard C, Shankarappa SA (2022). A brief review of in vitro models for injury and regeneration in the peripheral nervous system. Int J Mol Sci.

[66] Taylor AM, Blurton-Jones M, Rhee SW, Cribbs DH, Cotman CW, Jeon NL (2005). A microfluidic culture platform for CNS axonal injury, regeneration and transport. Nat Methods.

[67] Shi M, Majumdar D, Gao Y, Brewer BM, Goodwin CR, McLean JA (2013). Glia co-culture with neurons in microfluidic platforms promotes the formation and stabilization of synaptic contacts. Lab Chip.

[68] Sun X, Zhou Z, Fink DJ, Mata M (2013). Hspb1 silences translation of Pdz-Rhogef by enhancing Mir-20a and Mir-128 expression to promote neurite extension. Mol Cell Neurosci.

[69] Puttagunta R, Tedeschi A, Sória MG, Hervera A, Lindner R, Rathore KI (2014). Pcaf-dependent epigenetic changes promote axonal regeneration in the central nervous system. Nat Commun.

[70] Oh YM, Mahar M, Ewan EE, Leahy KM, Zhao G, Cavalli V (2018). Epigenetic regulator Uhrf1 inactivates rest and growth suppressor gene expression via DNA methylation to promote axon regeneration. Proc Natl Acad Sci U S A.

[71] Hu G, Huang K, Hu Y, Du G, Xue Z, Zhu X (2016). Single-cell RNA-Seq reveals distinct injury responses in different types of DRG sensory neurons. Sci Rep.

[72] Yan WT, Lu S, Yang YD, Ning WY, Cai Y, Hu XM (2021). Research trends, hot spots and prospects for necroptosis in the field of neuroscience. Neural Regen Res.

[73] Li XJ, Li CY, Bai D, Leng Y (2021). Insights into stem cell therapy for diabetic retinopathy: a bibliometric and visual analysis. Neural Regen Res.

[74] Bonizzato M, Pidpruzhnykova G, DiGiovanna J, Shkorbatova P, Pavlova N, Micera S (2018). Brain-controlled modulation of spinal circuits improves recovery from spinal cord injury. Nat Commun.

[75] Capogrosso M, Milekovic T, Borton D, Wagner F, Moraud EM, Mignardot JB (2016). A brain-spine interface alleviating gait deficits after spinal cord injury in primates. Nature..

[76] van den Brand R, Heutschi J, Barraud Q, DiGiovanna J, Bartholdi K, Huerlimann M (2012). Restoring voluntary control of locomotion after paralyzing spinal cord injury. Science..

